# Key contribution of eIF4H-mediated translational control in tumor promotion

**DOI:** 10.18632/oncotarget.5442

**Published:** 2015-10-15

**Authors:** Charlotte Vaysse, Céline Philippe, Yvan Martineau, Cathy Quelen, Corinne Hieblot, Claire Renaud, Yvan Nicaise, Aurore Desquesnes, Maria Pannese, Thomas Filleron, Ghislaine Escourrou, Malcolm Lawson, Robert C. Rintoul, Marie Bernadette Delisle, Stéphane Pyronnet, Pierre Brousset, Hervé Prats, Christian Touriol

**Affiliations:** ^1^ INSERM U1037, CRCT, Cancer Research Center of Toulouse, Toulouse, France; ^2^ Toulouse University, Paul Sabatier, Toulouse, France; ^3^ Department of Thoracic Surgery, Rangueil-Larrey Hospital, Toulouse, France; ^4^ Department of Pathology, CHU Rangueil, Toulouse, France; ^5^ Inserm US006 Crefre, Toulouse, France; ^6^ San Raffaele Scientific Institute, Milano, Italy; ^7^ Clinical trial office–Cellule Biostatistique Institut Universitaire du Cancer Toulouse, Toulouse, France; ^8^ Department of Respiratory Medicine, Broomfield Hospital, Chelmsford, Essex, UK; ^9^ Department of Thoracic Oncology, Papworth Hospital, Cambridge, UK; ^10^ Department of Pathology, Institut Universitaire du Cancer, Toulouse, France

**Keywords:** lung carcinoma, chemoresistance, translation initiation factor, eIF4H, IRES, helicase

## Abstract

Dysregulated expression of translation initiation factors has been associated with carcinogenesis, but underlying mechanisms remains to be fully understood. Here we show that eIF4H (eukaryotic translation initiation factor 4H), an activator of the RNA helicase eIF4A, is overexpressed in lung carcinomas and predictive of response to chemotherapy. In lung cancer cells, depletion of eIF4H enhances sensitization to chemotherapy, decreases cell migration and inhibits tumor growth *in vivo*, in association with reduced translation of mRNA encoding cell-proliferation (c-Myc, cyclin D1) angiogenic (FGF-2) and anti-apoptotic factors (CIAP-1, BCL-xL). Conversely, each isoform of eIF4H acts as an oncogene in NIH3T3 cells by stimulating transformation, invasion, tumor growth and resistance to drug-induced apoptosis together with increased translation of IRES-containing or structured 5′UTR mRNAs. These results demonstrate that eIF4H plays a crucial role in translational control and can promote cellular transformation by preferentially regulating the translation of potent growth and survival factor mRNAs, indicating that eIF4H is a promising new molecular target for cancer therapy.

## INTRODUCTION

One of the hallmarks of cancer cells is their ability to undergo rapid growth. This requires a sustained increase in protein synthesis and therefore cancer cell progression involves the dysregulation of translation, in particular that of specific transcripts that confer growth advantages [1, 2].

**Figure 1:**
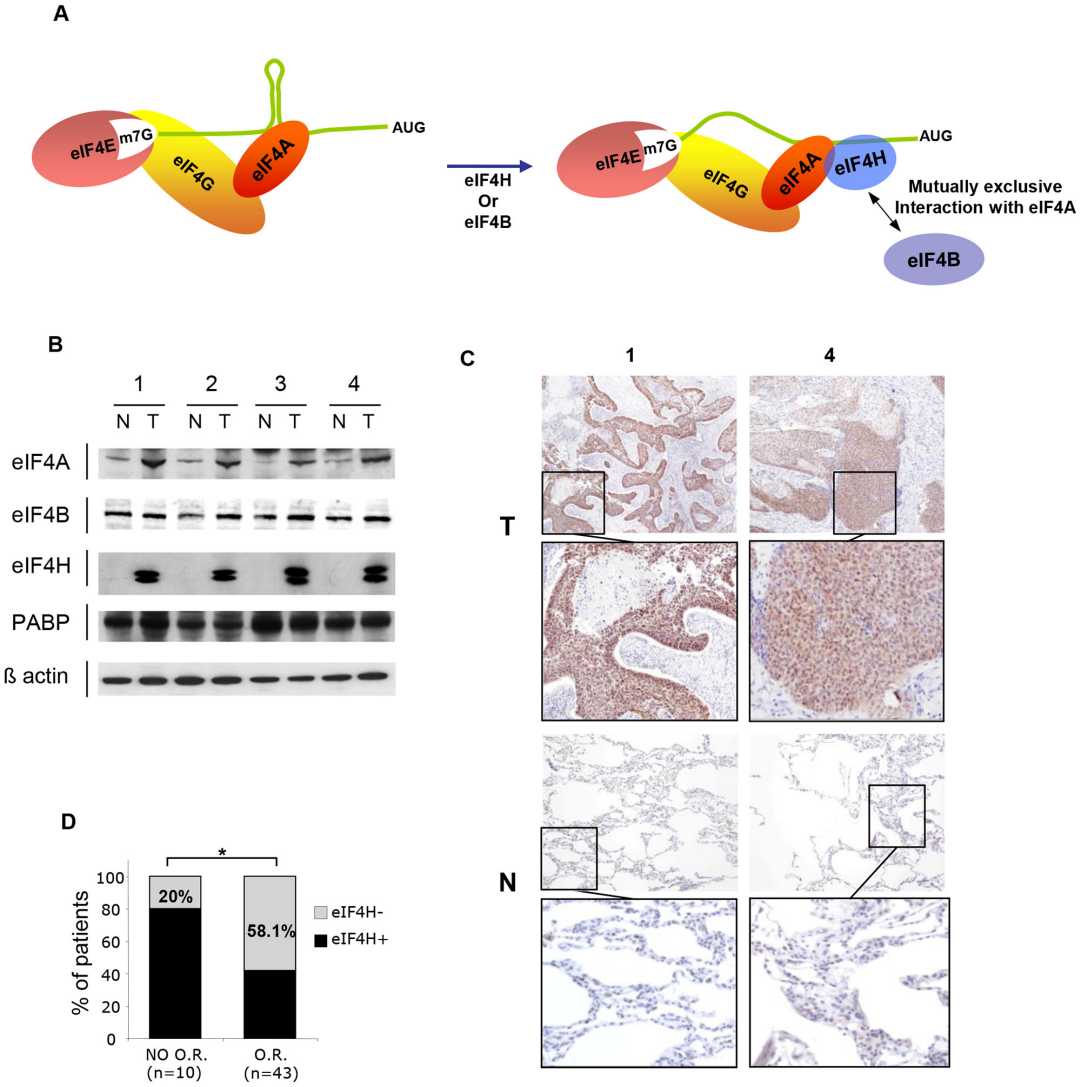
eIF4H expression in lung carcinomas **A.** Schematic model for cap-dependent initiation. eIF4E interacts with the mRNA 5′ cap structure and forms the eIF4F complex by association with the RNA helicase eIF4A and the scaffolding protein eIF4G. The helicase activity of eIF4A is stimulated by eIF4H or eIF4B (right-hand side). **B.** Western blot analysis of protein lysates prepared from 4 matched samples of lung carcinoma tumors (T) and adjacent non-tumoral tissues (N) Equal amounts of protein from each pair were resolved on SDS PAGE and immunoblotted with anti-eIF4A, eIF4B, PABP, eIF4H and β-actin (loading control) antibodies. **C.** eIF4H immunostaining of normal (N) and tumoral (T) tissues corresponding to samples 1 and 4 in (B). **D.** Objective Response (OR) to etoposide and cisplatin treatment based on the Response Evaluation Criteria in Solid Tumors (RECIST) of 53 patients with small cell lung carcinomas.

Translation initiation is dependent on the spatial and temporal interactions between many eIFs (eukaryotic translation Initiation Factors). First, a ribosome is recruited to the 5′ untranslated region (UTR) of the transcript. This involves the eIF4F complex which is composed of three initiation factors: the cap-binding protein eIF4E, the prototypical DEAD-box helicase eIF4A and the large scaffold protein eIF4G that is able to directly interact with the eIF4E and eIF4A proteins (Figure 1A) [3–5]. Once the eIF4F complex has been recruited, eIF4A unwinds the inhibitory RNA secondary structure within the 5′UTR (Figure 1A), increasing the ability of the 40 S ribosomal subunit to bind to the mRNA. By itself, eIF4A displays a weak ATP-dependent helicase activity, but this is enhanced through a functional interaction with either eIF4H or eIF4B [6–12]. Neither eIF4B nor eIF4H exhibits a helicase activity in the absence of eIF4A but they promote the ATPase [7, 13–15], RNA-binding [7, 10, 12, 15, 16], and helicase activities [12–14, 17] of eIF4A. This allows eIF4A to efficiently unwind the secondary structures in the 5′UTR, to promote the efficient scanning of the ribosome up to the start codon. eIF4A-mediated RNA unwinding appears to be necessary for ribosome recruitment even for mRNA with few secondary structures [17]. Recently, it was demonstrated that the eIF4A/eIF4H complex can repetitively unwind RNA hairpins by transitioning eIF4A between an active and inactive conformation using energy from ATP hydrolysis. The complex can be inactivated using a specific inhibitor that is able to lock eIF4A in its inactive conformation [18].

As a translation initiation factor eIF4H plays an important role within the cell, yet it has not been well-characterized compared to some of the other eIFs. eIF4H has two transcript variants as a result of alternative splicing, leading to the expression of two protein isoforms of 25 and 27 kDa. These share significant sequence homology with eIF4B. The function of eIF4B depends on its phosphorylation (on Ser422) by the ribosomal S6 kinase through a Rapamycin-sensitive pathway and several studies have shown the importance of eIF4B Ser422 phosphorylation for the initiation of cap-dependent translation [19, 20–22]. Interestingly, eIF4B and eIF4H hold homologous RNA recognition motif (RRM) domains, however eIF4H lacks the carboxy-terminal region containing the phosphorylation site, suggesting that eIF4H may be constitutively active. The eIF4H gene is located within the Williams-Beuren Syndrome critical genomic region. This pathology is an autosomal dominant disorder that results from a hemizygous deletion of sequences on chromosome 7q11.23, including the eIF4H gene region. The clinical outcome of this syndrome includes cardiovascular, renal, dysmorphologic and ophthalmic abnormalities as well as neurological and cognitive disorders. eIF4H-deficient knockout mouse harbors growth defects, body weight loss, brain abnormalities, altered neuronal morphology and several behavior anomalies. This demonstrates that eIF4H depletion may contribute to certain deficiencies associated with Williams-Beuren Syndrome [23].

Recent studies have implicated eIFs in the progression of various types of cancer. eIF4A was shown to be an oncogene in T-cell acute lymphoblastic leukaemia (T-ALL) where it is required for the translation of transcripts with 5′UTRs that can form G-quadruplexes [24]. In addition, eIF4B was reported to increase the synthesis of proteins associated with enhanced diffuse large B-cell lymphoma cell survival [25]. The 27 KDa eIF4H isoform was also found to be overexpressed in human colorectal and esophagus cancer tissues [26], where the silencing of only the long eIF4H isoform inhibited proliferation and induced apoptosis of colon cancer cells, suggesting that this isoform specifically contributes to cell proliferation and carcinogenesis. Nevertheless, the exact role of eIF4H in tumorigenesis and the molecular mechanisms involved are unknown. In this study, we have investigated the role of eIF4H in cellular transformation and its physiological role in mRNA translation both by overexpression of the two eIF4H isoforms in NIH3T3 cells and downregulation of the isoforms by RNA interference in lung cancer cells.

## RESULTS

### eIF4H isoform expression in lung tumors

We first investigated eIF4H expression in various tumor types using a high-density multiple organ tumor and normal tissue array, which contains 18 types of tumor alongside normal controls (MC5003, Biomax, US). Given that eIF4H was specifically highly expressed in many lung tumor tissue (Supplementary Figure S2A–S2D) but was undetectable or very low expressed in all normal lung tissue (Supplementary Figure S2E–S2G) we focus our study on lung tumors.

Alternative splicing of the eIF4H gene generates two transcript variants producing 25 kDa and 27 kDa protein products, thus we investigated their expression levels in lung tumors. For this purpose, total protein lysates were prepared from 12 matched samples of lung tumor (T) and adjacent normal tissue (N). We established that the expression levels of both eIF4H isoforms was significantly increased in lung carcinomas compared to corresponding healthy tissue (Figure 1B and Supplementary Figure S2H). The expression of eIF4A was also slightly increased in lung tumor patient samples, while there was no difference in the levels of eIF4B and PABP. eIF4H total protein expression in these tumors (Figure 1B, Supplementary Figure S2H) was confirmed by immunohistochemistry using histological sections of tumor (T) and adjacent normal tissue (N) (Figure 1C and Supplementary Figure S2I).

We then performed eIF4H immunohistochemical detection on a small cell lung carcinoma tissue microarray (TMA; described in [27]). Positive eIF4H levels were associated with a lack of objective response (OR) in patients that received etoposide and cisplatin doublet chemotherapy. Indeed, in the patient group with no OR to treatment based on Response Evaluation Criteria in Solid Tumors (RECIST) criteria, only 20% of samples did not express eIF4H, while 58.1% of patients in the group with OR showed no eIF4H staining (Figure 1D). These findings indicate that eIF4H expression may serve as a new molecular marker for predicting the response to etoposide and cisplatin therapy. No significant correlation between the expression of eIF4H and overall survival was found probably due to the lack of statistical power of this small cohort.

### Effect of eIF4H knockdown on drug-induced apoptosis and tumor progression

**Figure 2:**
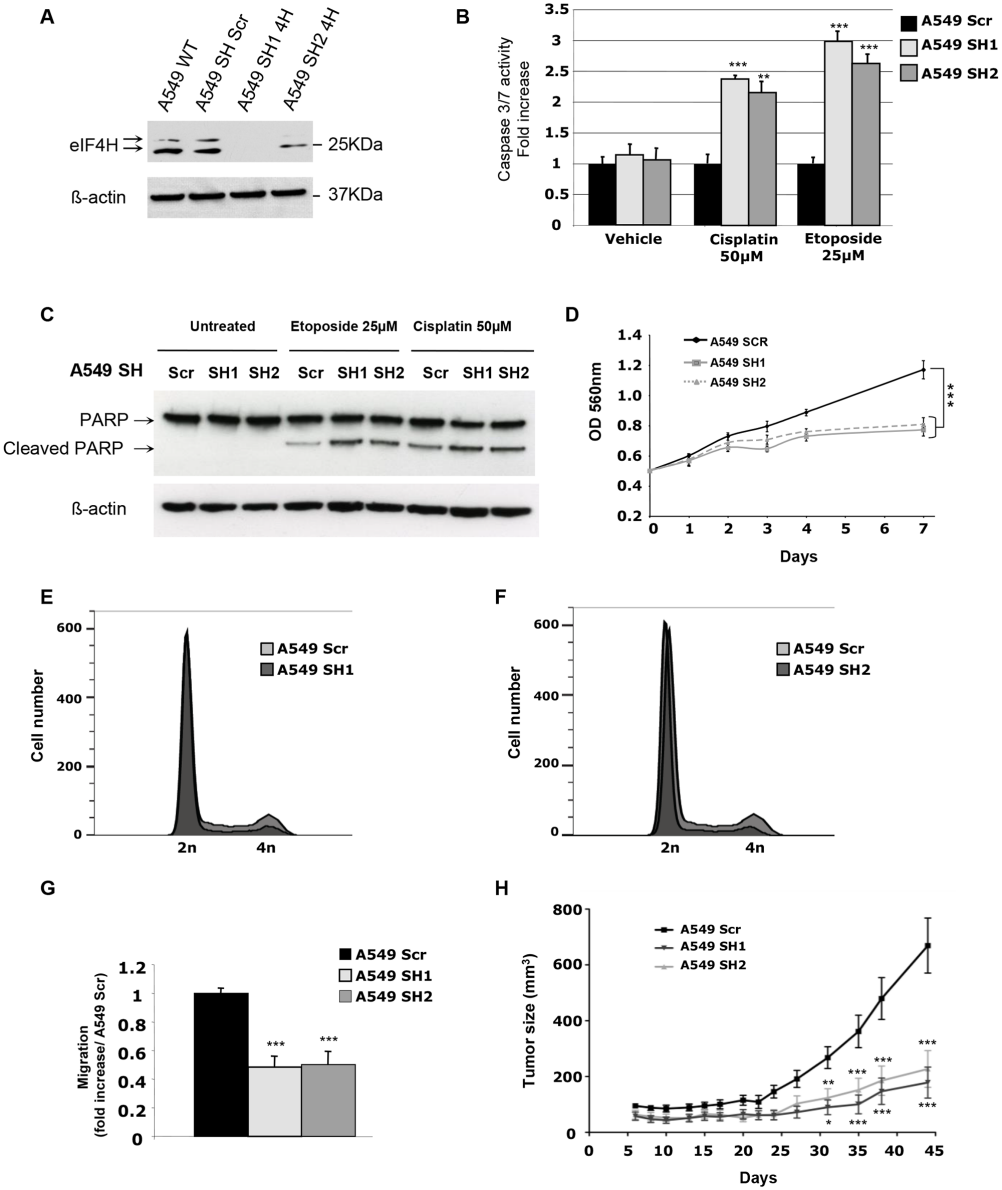
*In vitro* and *in vivo* characterization of the effect of eIF4H knockdown in A549 cells **A.** Expression analysis of eIF4H and β-actin (loading control) in either A549 wild type (WT) cells or stable clones expressing scrambled shRNA (sh SCR) or eIF4H-targeting shRNA (sh1-4H and sh2-4H). **B.** Caspase 3/7 activity induction after 8 hours treatment with etoposide (25 μM) or cisplatin (50 μM) in A549 cells expressing eIF4H (sh1 and sh2) or scrambled shRNA. **C.** PARP cleavage analysis by immunoblotting. A549 cells expressing eIF4H (sh1 and sh2) or scrambled shRNA were untreated or treated for 8 hours with etoposide (25 μM) or cisplatin (50 μM). Full length and typical PARP cleavage were detected. β-actin was used as a loading control. **D.** Cell proliferation of A549 cells stably expressing or scrambled shRNA under low serum conditions (0.5%) over 7 days using MTT. **E.** and **F.** Cell cycle analysis of A549 cells expressing eIF4H sh1 (E) or eIF4H sh2 (F) and scrambled shRNA was carried out using flow cytometry. **G.** Migration of A549 cells transfected with scrambled shRNA or eIF4H-targeting shRNA was measured in a Boyden chamber assay. Fold induction represent the average number of cells/field in the sh4H-expressing cells over control cells (Scr). **H.** Tumor volumes measured at indicated time points after subcutaneous injection of eIF4H-deficient or control A549 cells into 10 nude mice in each group. Error bars show SEM.

We evaluated the consequences of shRNA-mediated eIF4H depletion in A549 cells (lung carcinoma) and HeLa cells (cervical adenocarcinoma), by measuring drug-induced apoptosis, cell proliferation, migration and tumor growth. eIF4H knockdown (eIF4H-kd) was validated by western blotting, and two independent clones exhibiting a high silencing efficiency were selected for these studies (Figure 2A and Supplementary Figure S3A).

Given that eIF4H was highly expressed in lung carcinomas displaying resistance to chemotherapy, we first assessed the effect of eIF4H depletion on cisplatin or etoposide chemoresistance *in vitro* in A549 cells. As shown in Figure 2B, after 8 hours of cisplatin or etoposide treatment, eIF4H-kd cells displayed increased caspase 3/7 activity compared to control shRNA-transfected cells. Similar results were obtained for HeLa cells treated with cisplatin (Supplementary Figure S3B). We also tested an alternative apoptotic response pathway by using western blotting to examine poly(ADP-ribose) polymerase (PARP) cleavage. Compared to control cells, eIF4H knockdown resulted in increased PARP cleavage in A549 cells treated with cisplatin or etoposide (Figure 2C). We next investigated the effect of eIF4H depletion on cell proliferation and cell cycle progression. Upon eIF4H silencing, cell proliferation *in vitro* under low serum conditions (Figure 2D) was significantly reduced. Similar results were obtained with HeLa cells (Supplementary Figure S3C). eIF4H silenced cells showed a reduction in the percentage of cells in G2/M and accumulation of cells in G1 phase (respectively 82% and 82,7% versus 68,1% in control cells) indicating that eIF4H facilitates cell proliferation under low serum conditions (Figure 2E and 2F). Upon eIF4H silencing, cell migration (Figure 2G) was also significantly reduced.

Finally, the consequence of eIF4H depletion on lung tumor growth was assessed in a subcutaneous xenograft model. As shown in Figure 2H, eIF4H knockdown significantly inhibited A549 cell tumor growth compared with control groups (*P* < 0.001 at day 35). Similar results were obtained with HeLa cells (Supplementary Figure S3D).

Interestingly, upon immunofluorescence staining with CD31, we observed that angiogenesis was highly affected in engrafted A549 eIF4H knockdown cells compare to control A549 control cells (Supplementary Figure S4). Notably, density of CD31-positive vessels as well as pericyte coverage (α-SMA1+) was higher in control compare to eIF4H knockdown tumors.

Taken together, these data indicate that eIF4H expression not only enhances the resistance of tumoral cells to chemotherapeutic drugs but also promotes tumor growth and angiogenesis in nude mice.

### Effect of eIF4H isoforms on NIH3T3 cell proliferation, transformation, invasion properties, and resistance to drug-induced apoptosis

**Figure 3:**
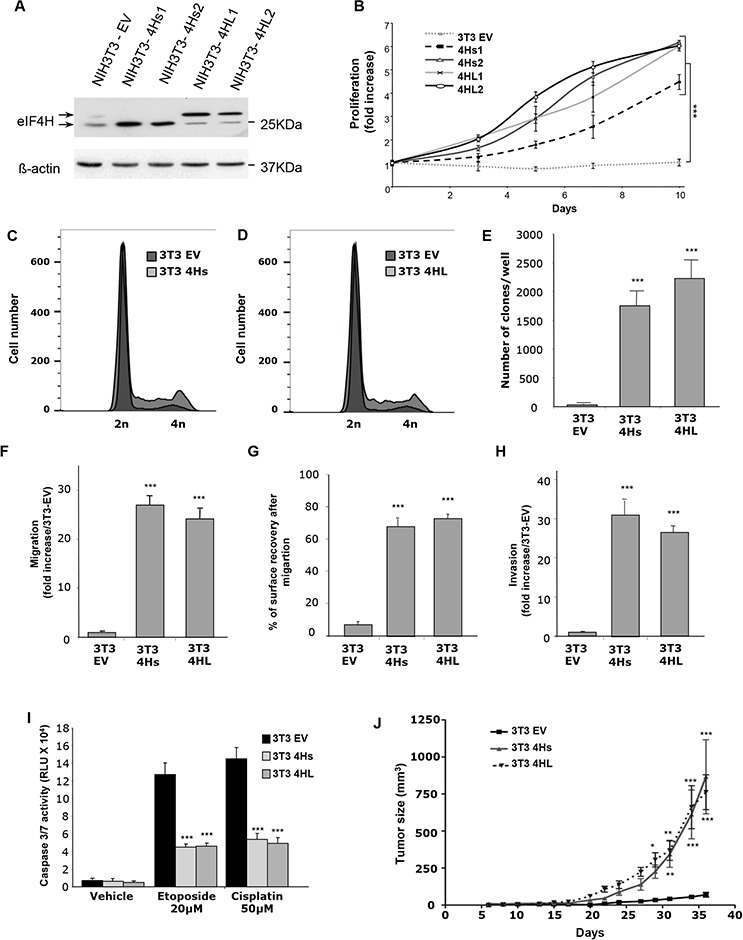
Consequences of eIF4H overexpression in NIH3T3 cells **A.** Expression analysis of eIF4H short isoforms (4Hs1 and 4Hs2) and long isoforms (4HL1 and 4HL2) transfected into NIH3T3 stable clones. The control was provided by NIH3T3 cells stably transfected with the empty vector (3T3 EV). Loading was normalized to β-actin. **B.** Proliferation of NIH3T3 stable clones overexpressing short (4Hs1 and 4Hs2) or long (4HL1 and 4HL2) eIF4H isoforms under low serum conditions (1%) for 10 days. **C.** and **D.** Cell cycle analysis of NIH3T3 stable clones overexpressing short (4Hs) (C) or long (4HL) (D) eIF4H isoforms under low serum conditions was carried out using flow cytometry. **E.** Colony formation of eIF4H-transfected NIH3T3 cell lines in soft agar. The number of clones in agar was determined after 25 days. **F.** Cell migration quantification of NIH3T3 stable clones in a Boyden chamber assay after 6 hours. The number of migrating cells was calculated by integrating 12 independent cellular fields (from 3 independent assays). **G.** Cell migration after wound breakage of a monolayer of NIH3T3 stable clones was determined by a cell restitution assay. Migration was quantified as the percentage of surface recovery after 8 hours. **H.** Invasive properties of NIH3T3 stable clones using an invasion chamber assay. After 24 hours incubation the invasive cell number was determined by crystal violet staining. Quantification of data was performed as in (E). **I.** Caspase 3/7 activity after 8 hours treatment with etoposide (20 μM) or cisplatin (50 μM) of control NIH3T3 cells or cells expressing the eIF4H isoforms. **J.** Tumorigenicity of NIH3T3 cells expressing the eIF4H isoforms. Tumor volumes were measured at the indicated time points after subcutaneous injection of NIH3T3 stable clones into 9 nude mice in each group. Error bars show SEM.

In order to study the individual contributions of each eIF4H splice variant on malignant transformation, we generated NIH3T3 cell lines stably-expressing either the longer 27 kDa isoform (4HL) or the shorter 25 kDa isoform (4Hs) under the control of the CMV promoter. After selection and screening for eIF4H expression by western blotting, four clones exhibiting about a 10-fold increased level of expression of the 27 kDa isoform (4HL1-4) or the 25 kDa isoform (4Hs1-4) were selected (Figure 3A and Supplementary Figure S5A). The elevated expression of both eIF4H splice variants stimulated cell proliferation under low serum conditions (1% FCS) (Figure 3B and Supplementary Figure S5B) but also increased the number of cells in G2/M and reduced the percentage of cells in G1 phase (respectively 63% and 67,8% versus 86,3% in control cells) (Figure 3C and 3D) and stimulated anchorage-independent cell growth based on cell colony formation in soft agar (Figure 3E and Supplementary Figure S5C).

We then evaluated the effect of eIF4H isoform overexpression on cell migration using both Boyden chamber or wound–healing assays. The number of migratory cells increased dramatically (more than 25 fold after 6 hours) in NIH3T3 cells expressing either eIF4H isoform (Figure 3F and Supplementary Figure S6A). We also observed that elevated levels of each eIF4H isoform in NIH3T3 cells stimulated wound closure compared to NIH3T3 control cells. Cells expressing the eIF4H isoforms were indeed able to migrate efficiently and cover more than 60% of the wounded area in 8 hours whereas control NIH3T3 cells were much less efficient in this process with less than 10% of the wounded area covered 8 hours (Figure 3G and Supplementary Figure S6B).

In order to evaluate the invasive properties of these eIF4H–transfected clones, we studied their capacity to cross a reconstituted basement-membrane matrix *in vitro*, using invasion chambers. NIH3T3 control cells were poorly invasive, while expression of the eIF4H isoforms was associated with invasive properties (30-fold increase compared to control cells) (Figure 3H and Supplementary Figure S6C).

The role of each eIF4H isoform in chemosensitivity was next evaluated. Clones expressing eIF4H splice variants or control cells were treated with cisplatin or etoposide for 8 hours. The overexpression of both eIF4H isoforms inhibited caspase 3 and 7 activity compared to control cells (Figure 3I).

Finally, eIF4H-expressing NIH3T3 cells were subcutaneously grafted into athymic nude mice. eIF4H expression significantly enhanced the growth of transplanted subcutaneous tumors, as shown in Figure 3J.

Taken together, these data demonstrate that eIF4H overexpression protects against drug-induced apoptosis *in vitro*, but also promotes cell proliferation, migration, invasion, and tumor growth. Elevated levels of each isoform share the same outcome for these phenotypes.

### eIF4H modulates the translation of specific mRNAs *in vitro*

**Figure 4:**
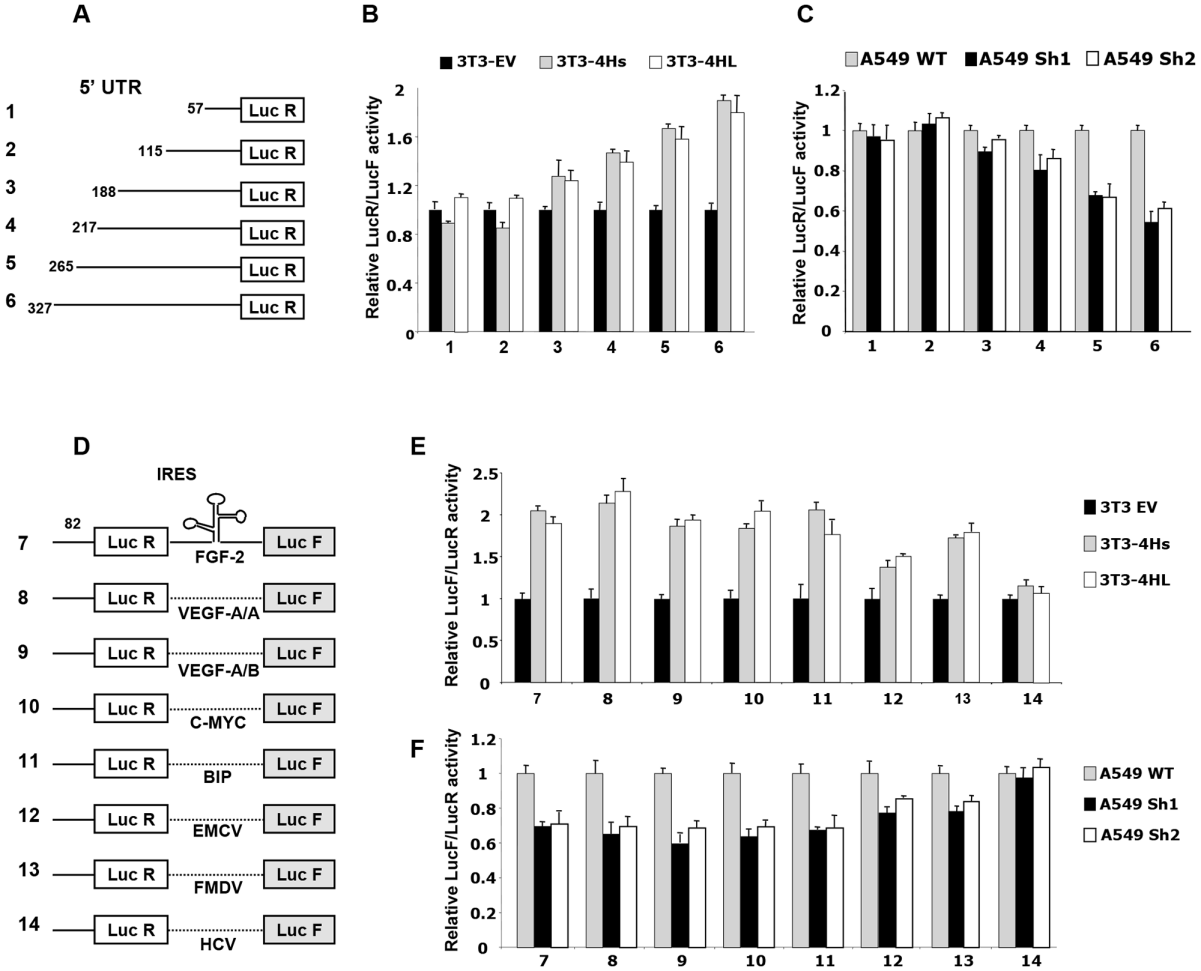
Effect of eIF4H on cap- and IRES-dependent translation **A.** Schematic representation of monocistronic constructs with different 5′UTR lengths. **B.** Ratio of luminescence from the experimental reporter (Renilla) to the control reporter (Firefly; PGL3 from Promega) after co-transfection of NIH3T3 cells expressing the short (4Hs) or long (4HL) eIF4H isoforms, normalized to NIH3T3 empty vector control cells (set as 1). **C.** Ratio of luminescence from the experimental reporter (Renilla) to the control reporter (Firefly; PGL3 from Promega) after co-transfection of A549 eIF4H knockdown cells (sh1 and sh2), normalized to the A549 scramble control cells (set as 1). **D.** Schematic representation of bicistronic constructs. IRESs cloned within the inter-cistronic region were either viral (EMCV, FMDV, HCV) or cellular (FGF-2, VEGF-A IRESA, VEGF-A IRESB, C-MYC and BIP). **E.** Ratio of luminescence from the IRES-dependent reporter (Firefly) to the cap-dependent reporter (Renilla) in NIH3T3 cells expressing the short (4Hs) or long (4HL) eIF4H isoforms, normalized to the NIH3T3 empty vector control cells (set as 1). **F.** Ratio of Firefly to Renilla luminescence in A549 eIF4H knockdown cells (sh1 and sh2), normalized to the A549 scramble control cells (set as 1).

Changes in the initiation of translation could signal either a change in global translational regulation or an altered translation of mRNAs encoding specific proteins. Usually, mRNAs encoding proteins whose expressions are highly regulated at the translational level have one or more structural elements within their 5′UTR that mediate translational control. Examples of such elements include Internal Ribosome Entry Sites (IRES), long or highly structured 5′UTRs, and binding domains for specific regulatory proteins. Thus, we investigated the ability of eIF4H to modulate the cap-dependent translation of a luciferase reporter mRNA where either the AUG start codon is preceded by synthetic 5′UTRs with different lengths (from 57 to 327 nucleotides) (Figure 4A), or where the mRNA undergoes cap-independent translation driven by several viral and cellular IRESs, using luciferase bicistronic constructs (Figure 4D). We analyzed luciferase expression for each construct after transient transfection into NIH3T3 cells expressing each eIF4H isoform or in NIH3T3 control cells, as well as in A549 and HeLa cells where eIF4H was stably downregulated. Unambiguously, data show that constructs with longer 5′UTRs had increased activity in NIH3T3 eIF4H-expressing cells compared to control cells (Figure 4B). In agreement with these results, eIF4H depletion in A549 and HeLa cells led to the translation inhibition of reporters bearing longer 5′UTRs (over 188 nucleotides) (Figure 4C and Supplementary Figure S7B).

Interestingly, the overexpression of each eIF4H isoform stimulated cellular IRES activity by about 2-fold (constructs 7–11, Figure 4E). EMCV and FMDV viral IRES activity was increased to a lesser extent (constructs 12 and 13, Figure 4E). As expected, the IRES activity of HCV, which was demonstrated to be eIF4A-independent [36], was not affected by increased eIF4H levels. The depletion of eIF4H in A549 cells also reduced cap-independent translation driven by FGF-2, VEGF-A, c-Myc, BIP, EMCV or FMDV IRES by about 40% (Figure 4F). Similar results were obtained with HeLa cells and eIF4H-knockout MEF cells (Supplementary Figure S7D and S7E). All these results demonstrate that eIF4H is able to specifically modulate the translation of both mRNA with long 5′UTRs and of IRES elements containing mRNAs. As observed earlier, the overexpression of both eIF4H isoforms share the same consequences on translational regulation.

### eIF4H promotes the expression of potent growth and survival factors

**Figure 5:**
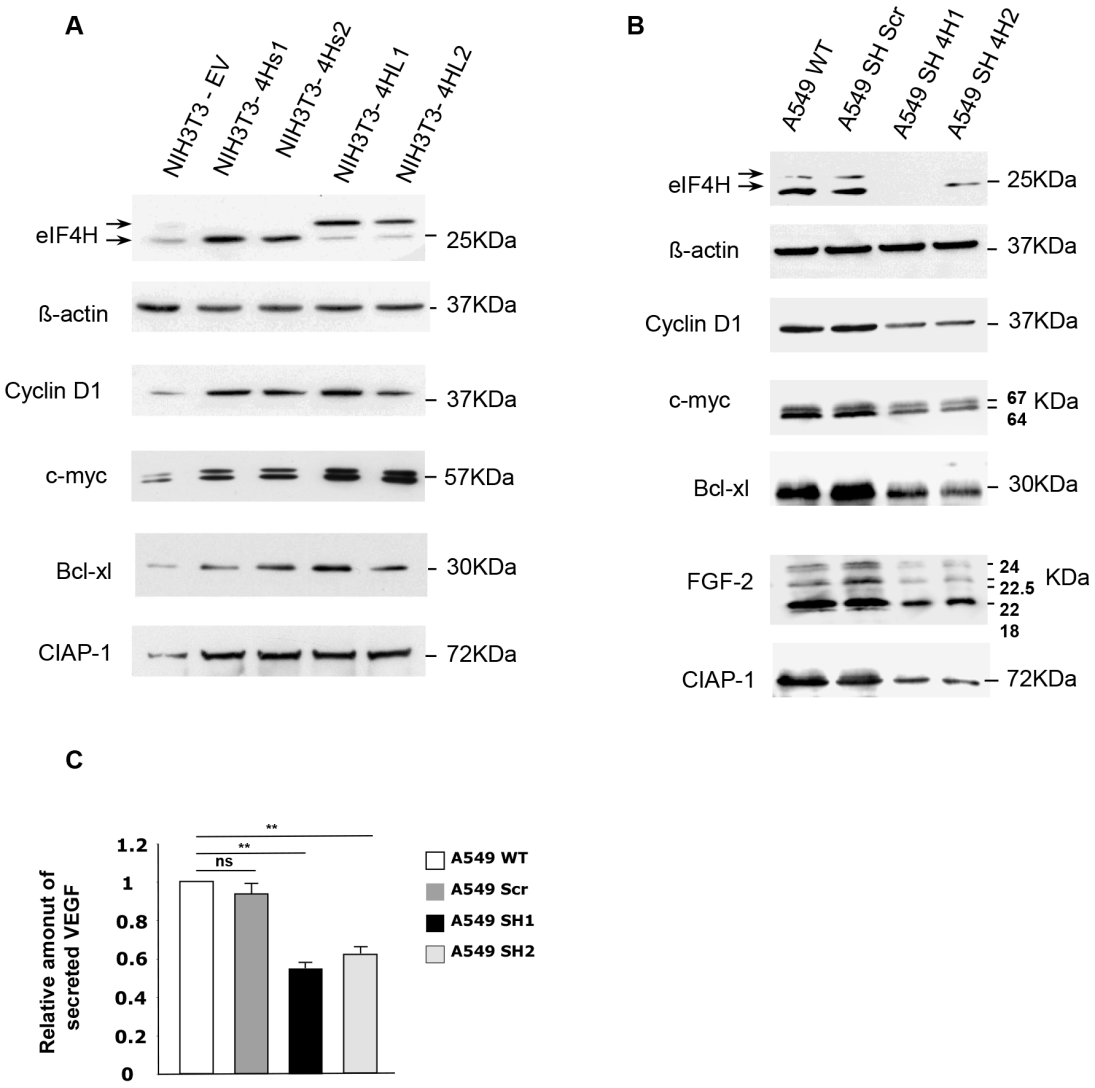
Effect of changes in eIF4H levels on the regulation of expression of genes involved in proliferation, apoptosis and cellular survival Western blots were performed to compare the expression of proteins encoded by mRNAs harboring complex 5′UTRs in both **A.** NIH3T3 control cells and clones stably-overexpressing eIF4H, and **B.** wild type A549 cells or A549 cells expressing scrambled (shScr) or eIF4H (sh1-4H, sh2-4H)-targeted shRNAs. The proteins detected as well as their molecular weights are indicated. The 5′UTRs length of these mRNAs are 210 nucleotides for cyclin D1, 526 nucleotides for c-myc, 367 nucleotides for Bcl-xL, 1400 nucleotides for CIAP1, 1038 nucleotides for VEGF-A, and 484 nucleotides for FGF-2. **C.** Relative amount of VEGF–A secreted by A549 wild type cells or cells expressing scrambled (Scr) or eIF4H (sh1 and sh2)-targeting shRNAs, as determined by an ELISA assay.

These results led us to hypothesize that eIF4H could be involved in the translation of a limited set of cellular mRNAs with complex 5′UTRs encoding growth factors, cell cycle regulators, or anti-apoptotic effectors. If this was the case, aberrant expression of these mRNAs would result in cell growth defects and deregulation of the apoptotic machinery. The expression of cellular mRNAs with complex 5′UTRs, including cyclin D1, c-Myc, FGF-2, Bcl-xl and CIAP-1 was examined by western blotting in NIH3T3 cells overexpressing the eIF4H isoforms or in eIF4H-kd A549 and HeLa cells. Data clearly showed that overexpression of eIF4H in NIH3T3 cells significantly increased the levels of cyclin D1 and c-Myc, two oncogenes both implicated in the stimulation of cell proliferation and in apoptosis-regulated process, as well as cIAP1 and Bcl-xL, two potent apoptosis inhibitors (Figure 5A). Interestingly, the two eIF4H isoforms elicited the same effect on the regulation of expression of these mRNAs. Conversely, the expression of cyclin D1, c-Myc, CIAP1 and Bcl-xl declined in eIF4H-depleted A549 (Figure 5B) or HeLa cells (Supplementary Figure S7A). We next investigated the effect of eIF4H on the expression of two human mRNAs containing G-quadruplex structures in their 5′UTRs which encode potent growth factors, namely FGF-2 [37] and VEGF-A [38]. Western blot analysis demonstrated that eIF4H-kd in A549 cells (Figure 5B) as well as in HeLa cells (Supplementary Figure S8), lowered the expression of the 4 endogenous FGF-2 isoforms. Finally, the VEGF protein levels in the culture medium were also decreased in eIF4H-kd A549 cells compared to control cells, as shown by ELISA (Figure 5C).

Taken together, these results suggest that eIF4H may contribute to tumor progression by promoting the expression of potent growth and survival factors.

### eIF4H stimulates the translation of mRNAs containing complex 5′UTRs

**Figure 6:**
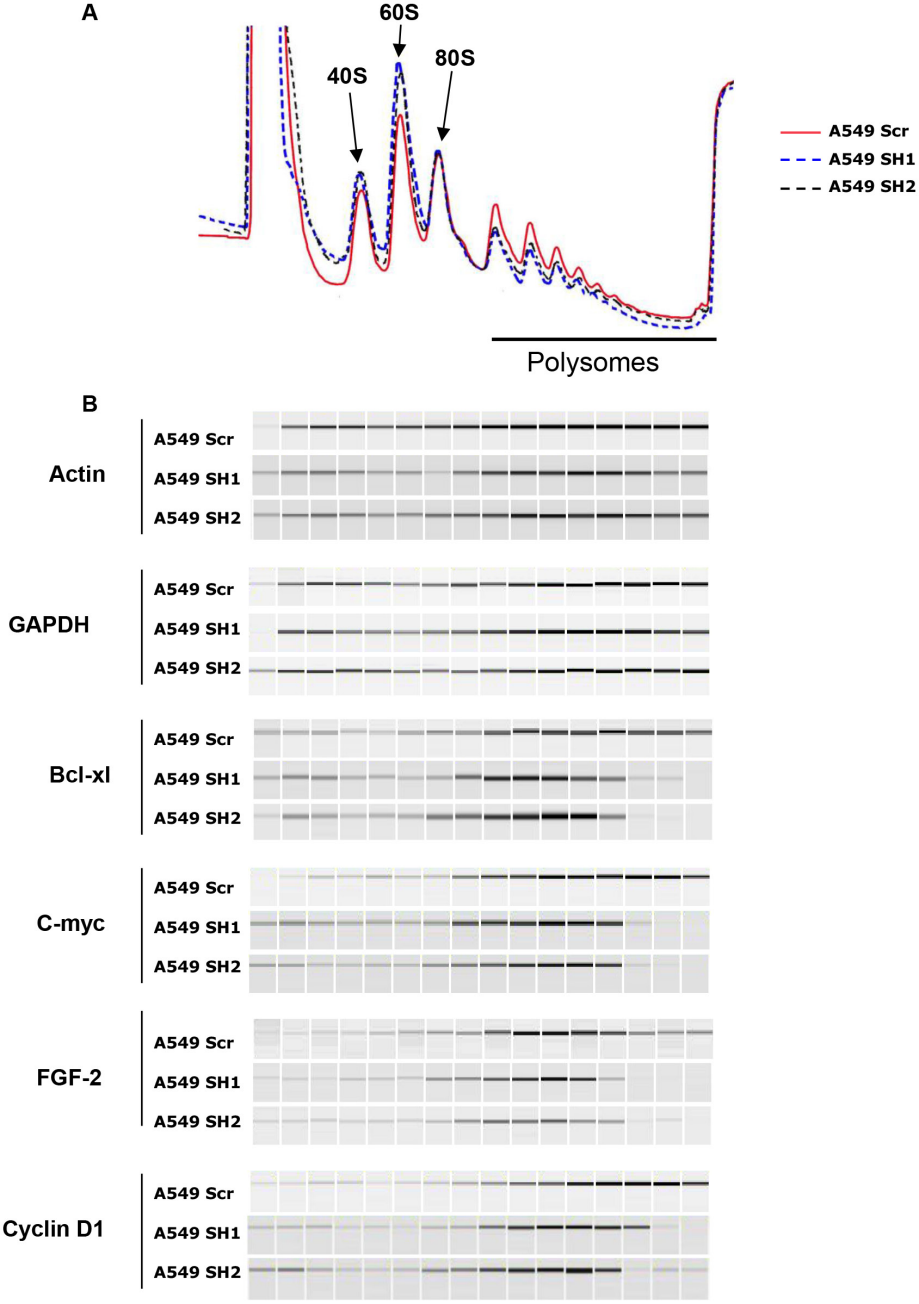
eIF4H stimulates the translation of mRNAs with structured 5′UTRs **A.** Polysomal profiles of A549 cells transfected with eIF4H (sh1 and sh2) or control shRNAs (Scr). **B.** Distribution of endogenous mRNAs (encoding Bcl-Xl, c-Myc, FGF-2, cyclin D1, actin and GAPDH) in sucrose density gradients from control (Scr) and eIF4H (sh1 and sh2)-silenced cells. PCR products were analyzed by capillary micro-electrophoresis on the Shimadzu MultiNA system.

To address whether eIF4H affected global translation or the translational efficiency of specific mRNAs we analysed polysome profiles from control versus eIF4H-kd A549 cells. Slight changes in the polysome profile (less polysomes and more free 40S and 60S subunits) were indicative of minor translation inhibition occurring in response to eIF4H knockdown (Figure 6A).

To examine the effects of changes in eIF4H levels on the translation of c-Myc, Bcl-xl, FGF-2 and cyclin D1 mRNA *in vivo*, we determined the distribution of these mRNAs in sucrose density gradients. In control cells, Bcl-xl mRNA sedimented predominantly with heavy polysomes whereas in eIF4H-silenced cells, it shifted to light polysomes, indicating a decrease in translation initiation of this mRNA (Figure 6B). Similar results were obtained with c-Myc, FGF-2 and cyclin D1 mRNAs. eIF4H depletion had a minimal effect on the polysomal distribution of actin and GAPDH mRNAs. These findings are in agreement with the observation that eIF4H depletion in A549 cells decreased c-Myc, FGF-2, Bcl-xl and cyclin D1 protein levels, while eIF4H overexpression in NIH3T3 cells resulted in increased expression of these proteins (Figure 5A and 5B).

In agreement with previous results obtained with reporter constructs, these data indicate that eIF4H preferentially stimulates the translation of mRNAs containing highly-structured 5′UTRs.

## DISCUSSION

Lung cancer has been associated with a number of genetic and epigenetic alterations but remains the major cause of cancer-related deaths throughout the world [39]. Therapeutic options are still limited and the prognosis remains poor even though the development of targeted therapies have improved lung cancer management. It has now become clear that genetic and epigenetic alterations are only the first layer of genetic reprogramming associated with tumor progression and that post-transcriptional regulation, including the control of mRNA translation, plays an important role in malignant transformation [40].

Protein synthesis is a major factor in determining cell phenotype and is a tightly regulated process that allows for a more rapid response than transcriptional control. Translation is primarily regulated at the ribosome recruitment step and the formation/activity of the eIF4F complex plays a central role in this mechanism. Tumor cells undoubtedly benefit from a hyperactive eIF4F complex, and this event is indeed observed in a wide range of cancers [41]. When activated, the eIF4F complex reprograms the cellular translational apparatus to amplify oncogenic signals and regulate neoplastic capabilities. The oncogenic activities of eIF4E have been demonstrated in multiple settings [42]. Similarly, eIF4G overexpression can drive the transformation of mouse cell lines [43], and eIF4A has been demonstrated to be an oncogene in T-cell acute lymphoblastic leukemia [24, 25]. Despite extensive interest in the eIF4E, eIF4G and eIF4A components, few studies have addressed the function of eIF4A cofactors, namely eIF4H and eIF4B, in cancer progression. Here we show that the two eIF4H isoforms are overexpressed in lung carcinomas and that the expression of this factor inversely correlates with the objective response to treatment.

We have demonstrated that each isoform of eIF4H acts as oncogene in NIH3T3 cells by increasing proliferation, migration and foci formation when grown on soft agar, and promoting chemoresistance. Moreover, eIF4H-expressing NIH3T3 cells are tumorigenic in nude mice. Our data also show that eIF4H stimulates the translation of mRNAs with either complex 5′UTRs or IRESs. A number of mRNAs enclosing these specific features encode growth factors, cell cycle or apoptotic machinery components. As a result, we have demonstrated that an alteration in eIF4H protein levels deeply affects the protein expression of potent cell growth and survival regulators or proteins involved in malignancy, such as cyclin D1, c-Myc, FGF-2, VEGF-A, Ciap1 and Bcl-xl. These results could account for the strong effect of eIF4H overexpression on the resistance to drug-induced apoptosis and tumor progression.

These data are in agreement with the biochemical function of eIF4H that specifically stimulates the eIF4A helicase activity. It has been postulated that eIF4A helicase activity significant affects the translation of individual mRNAs. When eIF4A helicase activity is low, it is thought that translation of mRNAs harboring short 5′UTRs is affected to a lesser degree than that of mRNAs possessing more extensive 5′UTR secondary structures. Indeed, dominant–negative eIF4A mutant proteins inhibit the translation of mRNAs possessing 5′UTR secondary structures, while the translation of mRNAs with short 5′UTRs is unaffected by inhibition of the RNA helicase [44, 45].

Recently it was shown that in breast cancer eIF4A preferentially stimulates translation of oncogenic genes, including cyclins, protein kinases, and mRNAs with G/C-rich 5′UTRs with potential to form G-quadruplexes [46].

Furthermore eIF4H stimulate IRES activity. IRES elements have also been mostly reported in mRNAs containing long 5′UTRs with a high GC content and an extensive predicted secondary structure. Interestingly, data from Sun and collaborators indicated that eIF4H could be targeted internally to stem/loop structures, in combination with eIF4A [12]. This interaction, which induces rapid mRNA remodeling, could explain the stimulation of IRES activity by increasing the recruitment of the initiation complex, or IRES Trans Acting Factors (ITAFs). These data have been strengthened by other studies using eIF4A inhibitors. These molecules were shown to induce both cell cycle arrest of adult T-cell leukemia by decreasing cyclin D1 expression levels, and apoptosis by decreasing the expression levels of Bcl-xl and other anti-apoptotic factors [47, 48], but also to inhibit cancer cell proliferation, and suppress cap- or IRES-driven translation without affecting HCV IRES-mediated translation [49]. eIF4H-silencing experiments displayed identical results, consistent with published evidence demonstrating that eIF4H stimulates eIF4A activity [6–8, 12, 15].

One must note that eIF4A is a very weak helicase by itself but its activity is enhanced upon mutually-exclusive interactions with eIF4B or eIF4H [6–12]. It is well-known that the mTOR signaling pathway results in the phosphorylation and activation of eIF4B by the ribosomal kinase S6K. Thus, nutrient starvation, osmotic stress, heat shock, ROS and DNA damage, which are known to decrease S6K activity, will cause the dephosphorylation of eIF4B and the inhibition of translation of highly-structured mRNAs [20, 21, 50, 51]. As previously mentioned, eIF4H is shorter than eIF4B but shares significant sequence homology in the RRM. However it lacks a large carboxy-terminal region encompassing the phosphorylation site (Ser422). We therefore envisage a model in which eIF4H is constitutively active and/or overexpressed leading to the expression of a subset of regulatory and stress response genes, while housekeeping genes remain unaffected. This hypothesis is supported by the fact that eIF4H knockdown inhibits the proliferation of A549 or HeLa cells under low serum culture conditions (Figure 2D and Supplementary Figure S3C). Moreover, eIF4H has been demonstrated to be under the transcriptional control of NF-kB [52], thought to be part of a stress response that is activated by a variety of stimuli including UV, serum starvation, and ER stress [53–55].

During tumor development and progression, hypoxia, acidosis, glucose depletion and a lack of other nutrients are caused by a combination of defective perfusion, abnormal tumor vasculature and uncontrolled proliferation of cancer cells, leading to eIF4B dephosphorylation [56]. Under these conditions, constitutively-active eIF4H could stimulate the translation of transcripts with complex 5′UTRs, conferring a relevant advantage to cancer cells for tumor growth, progression and resistance to chemotherapy. Nevertheless, it is clear that eIF4H has differential effects on the synthesis of proteins involved in the resistance to chemotherapy and lung tumor progression, and that these effects could represent a novel approach to lung carcinoma intervention.

eIF4H overexpression and its contribution to tumor growth are certainly not restricted to lung carcinoma. Indeed, eIF4H is detected in 93% of cancers (http://www.proteinatlas.org/ENSG00000106682/cancer), suggesting that eIF4H-mediated reprogramming of gene expression might be a general mechanism in tumoral development and a possible new therapeutic target.

## MATERIALS AND METHODS

### Human tissue samples

We prospectively recorded 12 patients with clinico-pathological data (Table 1), who underwent surgery for lung carcinoma at Rangueil Hospital (Toulouse, France) from 2007 to 2008. Tissues samples (tumoral tissues and corresponding non-tumoral counterpart) were obtained from pulmonary lobectomy or pneumonectomy specimens during surgery and were immediately frozen in liquid nitrogen and stored at −80°C. The protocol had local ethical committee approval. Consents were obtained from patients before surgery. Tissue microarray consisting of 184 cases of SCLC was previously described [27]. All cases were diagnosed at Papworth Hospital (Cambridge, UK) between 1998 and 2005. They were identified from hospital records and the formalin fixed, paraffin-embedded biopsy tissue samples were retrieved from the pathology department store along with their associated histology slides [27]. Clinical data were available for 53 of these patients that received etoposide and cisplatin doublet chemotherapy.

**Table 1:** Clinicopathologic characteristics of 12 patients with lung carcinoma

Patient	Gender	Surgical procedure	TNM	Stage	Histilogical type	Tumor size (cm)	Tumor extension	Positive lymph nodes
1	M	right pneumonectomy	yT2N1	IIB	adenocarcinoma	8	none	intrapulmonary
2	M	right upper lobectomy	pT2N2	IIIA	squamous cell carcinoma	3.5	none	mediastinal
3	M	right lower lobectomy	pT2N0	IB	squamous cell carcinoma	3.8	none	none
4	M	left upper lobectomy	pT3	IIB	squamous cell carcinoma	6	pleura	none
5	M	left pneumonectomy	pT2N1	IIB	squamous cell carcinoma	6	none	intrapulmonary
6	M	left upper lobectomy	pT2N0	IB	squamous cell carcinoma	4.5	none	none
7	M	left pneumonectomy	pT1(LUL) pT2(LLL) N0	IA and IB	squamous cell carcinoma (LUL) + adenocarcinoma (LLL)	1.5 (LUL) / 4 cm (LLL)	visceral pleura	none
8	M	right upper lobectomy	pT2N0	IB	Epidermoid carcinoma	3.5	none	none
9	F	left pneumonectomy	pT2N2	IIIA	adenocarcinoma	9	visceral pleura	mediastinal
10	M	left pneumonectomy	pT3N0	IIB	squamous cell carcinoma	5	mediastinal pleura	none
11	M	right lower lobectomy	pT2N0	IB	adenocarcinoma	4.8	none	none
12	F	right upper lobectomy	pT2N0	IB	adenocarcinoma	7	none	none

For each patient, numbered from 1 to 12, the relevant clinicopathologic characteristics, including their sex, surgical procedure, TNM classification, stage, histological type, size and extension of the primary tumors, and the presence of positive lymph nodes are shown. LUL (Left Upper Lobe) and LLL (Left Lower Lobe).

### Protein extraction and western blotting

Frozen tissue samples were pulverized with “Mikro-Dismembrator” (Sartorius, Aubagne, France) and resuspended in lysis buffer. Cells were scraped off into phosphate-buffered saline and lysed. Western blotting was performed as previously described [28]. Membranes were probed with antibodies against eIF4H, eIF4B and eIF4A (Ab, 1:1000, Cell Signaling Technology, Danvers, MA), CIAP-1 (Ab, 1: 2000, R&D Systems, Mineapolis, MN), Cyclin D1 (mAb, 1:1000, Cell Signaling Technology, Danvers, MA), c-Myc (mAb, 1: 500, Cell Signaling Technology, Danvers, MA), BCL-xL (mAb, 1:1000, Cell Signaling Technology, Danvers, MA), FGF-2 (Ab, 1: 200, Santa Cruz Biothechnology, sc-79, Santa Cruz, CA) PABP (mAb, 1: 500, Santa Cruz Biothechnology, sc-32318, Santa Cruz, CA). Primary antibody incubation was followed by incubation with a horseradish peroxidase-conjugated secondary antibody (anti-mouse dilution 1:5000, P8547, Sigma-Aldrich, Lyon, France or anti-rabbit dilution 1:5000, P9795, Sigma-Aldrich, Lyon, France). Proteins were visualized using a chemiluminescence ECL kit (RevelBIOt Plus, Ozyme, Saint-Quentin-en-Yvelines, France). The signal was normalized using anti-β–actin (mAb dilution 1: 5000, AC-15, Sigma-Aldrich, France).

### Immunohistochemical assay

The immunohistochemical analysis was performed with the same protocol for both TMA and human tissue samples. Slides were deparaffinised with xylene and rehydrated with ethanol. Antigenic retrieval was processed by submerging with the kit EnVision FLEX Target Retrieval Solution (Dako) on PT Link Dako. Between every step, a washing solution was used (EnVision FLEX Wash Buffer, Dako). Sections were then treated to block endogenous peroxidase activity and non-specific sites with the kit EnVision FLEX Peroxidase-Blocking Reagent (Dako, Denmark) during 5 min at room temperature.

The primary monoclonal anti-eIF4H antibody (anti-rabbit, 1:1000, Cell Signaling Technology, Danvers, MA) was incubated for 20 min at room temperature followed by incubation with the labeled polymer (Envision Flex Horse Radish Peroxidase, Dako), for 20 min. Staining is completed by a 10 minute incubation at room temperature with 3,3′-diaminobenzidine (DAB)+ substrate-chromogen (EnVision FLEX Substrate Working Solution, Dako).

All slides were counterstained with hematoxylin to visualize the nuclei (EnVision FLEX Hematoxylin) during 5 min room temperature.

Scoring was performed blinded to the clinical data relating to the case, simultaneously by two observers. The staining was homogeneous in tumoral cells. As we observed a good correlation between the percentage of positive tumoral cells and intensify, we scored in 2 groups: negative or positive for the expression of eIF4H.

### Cell culture

The HeLa (obtained from ATCC n°. CCL2TM), A549 (ATCC n° CCL-185) and NIH 3T3 (obtained from European Collection of Cell Cultures N° 93061524) cell lines were maintained in DMEM 1g/l glucose supplemented with 10% FCS, 1% glutamine, 0.1% gentamycin and incubated in a humidified atmosphere of 5% CO_2_ at 37°C.

### Establishment of NIH3T3, A549 and HeLa cellular models

Stable transfected NIH3T3 clones were obtained by JetPEI (Polyplus) transfection with two eIF4H-expressing vectors (see below). Cultures were maintained for 2 weeks in the presence of 1 mg/ml G418, then 36 clones from each transfection experiment were picked and transferred onto 24-well plates before cultivation in larger dishes and eIF4H expression analysis.

Lentiviral constructs targeting eIF4H (TRCN 0000153576, corresponding to the sequence CCGGGA TCTCAGCATAAGGAGTGTACTCGAGTACACTCCTT ATGCTGAGATCTTTTTTG –sh4H1; and TRCN0000 275667, corresponding to the sequence CCGGGATCTCA GCATAAGGAGTGTACTCGAGTACACTCCTTATGCTG AGATCTTTTTG - sh4H2) and non-targeting shRNA vectors (SHC002V, shScr) were purchased from Sigma. After transduction, A549 and HeLa cells were selected using 1 μg/ml puromycin.

### Plasmid constructions

The sequences encoding the two splice variants of eIF4H were PCR amplified using the primer pair 4HATG-F (AAGCTAGCTATCCATGGCGGACTTCGACACCTACG ACG) and 4HSTOP-R (AAAAGATCTCCCGGGA GCTC TCATTCTTGCTC CTTTTGAACGAC), with cDNA from HeLa cells as template. PCR products were digested by NheI and BglII and inserted into pCEN [29] digested by SpeI and BamHI in order to create pC4HsEN and pC4HLEN plasmids encoding respectively the short and the long eIF4H isoforms upstream of an EMCV-NEO cassette. Constructs were confirmed by sequencing and used to stably transfect NIH3T3 cells. Reporter vectors with long 5′UTRs were derived from a modified pRL-CMV (Promega) presenting a HA tag at the 5′ end of the luciferase coding region [30]. To generate PRL27, a deletion was performed between the NheI and HindIII sites using the Quikchange XL site-directed mutagenesis kit (Stratagene) and the oligonucleotides DEL1-F (5′GTTTAGTGAACCGT CAGATCACTAGAAGCTTGCTAGCCACCATGGCTT ACCCCG 3′) and DEL1-R (5′GGGTAAGCCATGGT GGCTAGCAA GCTTCTAGTGATCTGACGGTT CACT AAAC3′). Oligonucleotide Del1-F introduces a NcoI site at the luciferase AUG initiation codon. NcoI and HindIII digestion of pRL27 and double strand oligonucleotide insertion generated reporter constructs harboring a variable 5′UTR from nucleotides 57 to 327. The 17 nucleotides region upstream of the luciferase AUG start codon remained unchanged in all vectors. The resulting plasmids, namely pRL57, pRL115, pRL188, pRL217, pRL265 and pRL327, were checked by DNA sequencing. The sequences, folding and free energy of the thermodynamic prediction of the variable 5′UTRs are provided in Supplementary Figure S1. The bicistronic vectors used in this study have been previously described and validated [31, 32].

### Plasmid transfection

A549, HeLa or NIH3T3 cells were seeded into 24-well dishes 1 day prior to transfection and allowed to grow to 50% confluence. According to the manufacturer's instructions, 250 ng of each monocistronic or bicistronic reporter plasmid was transfected into the cells with the JetPEI reagent (Polyplus Transfection Illkirch, France) in 150 mM NaCl. Either the pRL-CMV control vector expressing Renilla luciferase, or the pGL3 firefly luciferase reporter vector (50 ng) were co-transfected into cells. This second reporter gene allowed the expression to be normalized for calculation of transfection efficiency.

### Migration assay

Cell migration was measured using the Boyden chamber assay (8 μm pore size transwells, Corning, Cell Biolabs, CBA-100-C, San Diego, CA). Cells (2.5 × 10^4^) in 0.5% FCS DMEM were seeded in the upper well. The lower chamber was filled with 10% FCS media. After 6 or 24 h, the non-migratory cells on the upper surface were removed with a cotton swab, and cells that had migrated to the lower filter were fixed and stained with 0.5% crystal violet. Migratory cells were counted under the light microscope. Each assay was performed in duplicate and three independent experiments were performed.

### Invasion assay

Invasion assay was assessed using 8 μm membrane pores coated with Matrigel Matrix (BD BioCoat Matrigel Invasion Chamber, BD biosciences, Franklin Lakes, NJ USA). 5 × 10^4^ NIH3T3, A549 or HeLa cells were suspended in 10% FCS DMEM, placed in the upper chamber and cultured for 24 h at 37°C. The cells that had invaded to the lower surface of the filter inserts were stained with crystal violet prior to cell density evaluation. Each assay was performed in duplicate and three independent experiments were carried out.

### Anchorage-independent growth assay

5 × 10^4^ NIH3T3 cells resuspended in DMEM containing 0.33% Noble agar (Gibco) were seeded onto 6 cm dishes over a 0.5% Noble agar lower coat [33]. Cells were fed every 3 days and colony number was scored after 25 days by 3-(4.5-dimethylthiazol-2-yl)-2.5-diphenyltetrazolium bromide (MTT) staining.

### Proliferation assays

Control NIH3T3 cells and stable clones overexpressing either the short eIF4H isoform (4Hs1 and 4Hs2) or the long isoform (4HL1 and 4HL2) were seeded in 96-well plates (800 cells) and cultivated under low serum conditions (1% FCS) for 10 days. HeLa or A549 cells were seeded in 96-well plates (1000 cells) and cultivated in low serum medium (0.5% FCS) for 5 days. Viable cell number was determined on the basis of mitochondrial conversion of MTT to formazine. Cells were incubated with MTT for 2 h at 37°C and the crystals were solubilized in dimethyl sulfoxide for 1 hour at RT. Optical density was read at 560 nm (Asys, Serlabo Technologies, Entraigues sur la Sorgue, France).

### Wound healing assay

Migration (wound healing) was performed using the CytoSelect™ 24-Well Wound Healing Assay (Cell Biolabs, San Diego, CA), according to the manufacturer's instructions. Briefly, NIH3T3 cells (2.5 × 10^5^) were seeded in wells with inserts and cultured until a monolayer was formed (24 h). After insert removal, migration was monitored for 3, 8 or 20 h. All experiments were repeated at least three times and data are presented as mean values.

### Cell cycle analysis

Cells were washed with phosphate-buffered saline and fixed in cold 70% ethanol overnight at −20°C. Cells were then washed twice with phosphate-buffered saline −0.1% bovine serum albumin and once with phosphate-buffered saline then labeled using propidium iodide staining for 30 min (Invitrogen). Cell cycle distribution was evaluated by fluorescence analysis on a MACSQuant VYB (Miltenyi Biotec). Cell doublets were excluded and 10000 events per condition were analyzed.

### Quantification of VEGF by ELISA

The amount of VEGF in the A549 cell culture medium was measured using ELISA kits specific for VEGF (R&D Systems, Minneapolis, MN, USA), according to the manufacturer's instructions.

### Drug treatments / Apoptosis assay

To test the effect of different drugs, cells were plated into 96-well plates and incubated with varying concentrations of drugs for 8 h. NIH3T3 and A549 stable clones were treated with a range of 10 to 100 μM for cisplatin and 5 to 50 μM for etoposide. HeLa cells were incubated in the presence of 100 μM cisplatin, 20 μM etoposide. Apoptosis was evaluated by measuring caspase-3/7 activity using the Caspase-Glo 3/7 Assay (Promega, Lyon, France). An incubation of 1 h at 37°C was performed after addition of the Caspase-Glo 3/7 reagent. Luminescence was recorded using the Centro LB 960 (Berthold Technologies, Bad Wilbad, Germany) and the background luminescence was subtracted from experimental values.

### Luciferase activity

The two luciferase activities (LucR and LucF) were measured in NIH3T3, A549 or HeLa cell extracts using the Dual Luciferase kit (Promega, Lyon, France) as previously described [34].

### Sucrose-gradient fractionation, polysome-associated RNAanalysis and RT–PCR

Sucrose density-gradient centrifugation, used to separate ribosomes into polysomal and subpolysomal forms, were performed essentially as described previously [35]. Briefly, extracts from A549 cells were prepared by lysis at 4°C in extraction buffer (10 mM Tris–HCl, pH 8.0, 140 mM NaCl, 1.5 mM MgCl2, 0.5% Nonidet-P40 and 500 U/ml RNAsin), and nuclei were removed by centrifugation (12 000g, 10 s, 4°C). The supernatant was supplemented with 20 mM dithiothreitol, 150 μg/ml cycloheximide, 1 mM phenylmethylsulfonyl fluoride and centrifuged (12 000g, 5 min, 48°C). The supernatant was layered onto a 12 ml linear sucrose gradient (15–40% sucrose (w/v) supplemented with 10 mM Tris–HCl, pH 7.5, 140 mM NaCl, 1.5 mM MgCl2, 10 mM dithiothreitol, 100 μg/ml cycloheximide) and centrifuged in a SW41Ti rotor (Beckman, Villepinte, France) for 2 h at 160 000g, 4°C, without braking. Fractions of 750 μl were collected using an ISCO collector and UV optical unit type 11 (Lincoln, NE). The data were acquired using an analog-to-digital converter USB-1208 device and Tracer-DAQ software (Measurement Computing Inc.). Each fraction was digested with 100 μg of proteinase K in 1% SDS and 10 mM EDTA (30 min, 37°C). RNAs were then recovered by phenol–chloroform–isoamyl alcohol extraction followed by ethanol precipitation. Pellets were washed with 70% ethanol pre-stored at 20°C, air-dried and resuspended in appropriate volumes of RNAse-free water. RNAs were analyzed by electrophoresis on 1.2% agarose gels. Reverse transcription was performed on 1/10 of each fraction using the RevertAid First Strand cDNA Synthesis Kit (Fermentas) with a random hexamer according to the manufacturer's recommendations. The resulting cDNA fragments were PCR-amplified using the Phusion Taq DNA polymerase (Finnzymes) and specific primers.

### Tumor growth

Animal experiments were conducted in accordance with the European directive, and with approval from the Regional Ethics Committee of Midi-Pyrénées for Animal Experimentation. NIH3T3 (2 × 10^6^), A549 or HeLa cells (1 × 10^6^) were injected subcutaneously into the flank of 6-week-old female nude mice (Janvier, Le Genest-Saint-Isle, France). Animal body weight and tumor volumes were measured 3 times per week until the end of the experiment.

### Statistical analyses

All experimental data are expressed as mean ± SEM and were analyzed by ANOVA followed by Student's *t*-test. Differences were considered significant at values of *P* < 0.05 (* : *P* < 0.05; ** : *P* < 0.01; *** : *P* < 0.001). For clinical data, categorical variables were summarized as the frequency and percentage, Comparison between groups were performed using chi-square test or fisher exact test for qualitative variable.

## SUPPLEMENTARY FIGURES


