# Supplementary material for: Key contribution of eIF4H-mediated translational control in tumor promotion
Source: Oncotarget. 2015 Oct 15;6(37):39924–40. doi: 10.18632/oncotarget.5442 (PMC4741870; doi:10.18632/oncotarget.5442)
Supplement: Supplementary file 1 [file oncotarget-06-39924-s001.pdf]

SUPPLEMENTARY FIGURES

VECTOR NAME	SEQUENCE 5' ⇒ 3' (encompassing AUG)	FREE ENERGY	PREDICTED STRUCTURE
PRL57	CAGATCACTAGAAGCTATCCTAGAG ACATAGCGCATTAGCCAAGCTTACT AGCCACCATG	-10,15 kcal/ mol	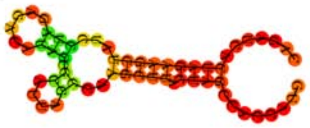
PRL115	CAGATCACTAGAAGCTATCCTAGGA ACTCTCAATTGCCACTCAGATTCTA GAGACATAGCGCATTAGCCAAGCTA ACTACAGTACATCCACTATAGACAAA GCTTCCTAGCCACCATG	-22,23 kcal/ mol	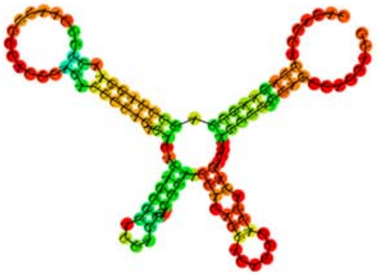
PRL188	CAGATCACTAGAAGCTATCCTAGGA ACTCTCAATTGCCACTCAGATTCTA GAGACATAGCGCATTAGCCAAGCTA ACTACAGTACATCCACTATAGACAAA TCTTCGAACTAACTTCCTAGCTAGG CATCATCAAGCTAACTACTAACTTCC TAGCTAGGCATCTACTAACTAAGCTT CCTAGCCACCATG	-40,21 kcal/ mol	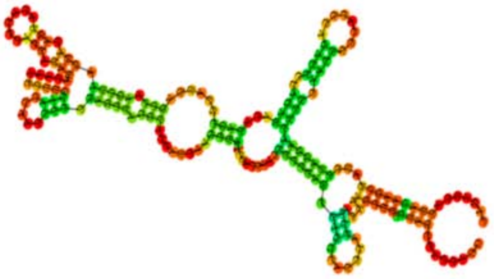
PRL217	CAGATCACTAGAAGCTATCCTAGGA ACTCTCAATTGCCACTCAGATTCTA GAGACATAGCGCATTAGCCAAGCTA ACTACAGTACATCCACTATAGACAAA TCTTCGAACTAACTTCCTAGCTAGG CATCATCAAGCTAACTACAGTACATC CACTATAGACAGATCTTCGAACTAA CTTCCTAGCTAGGCATCTACTAACTA AGCTTCCTAGCCACCATG	-44,92 kcal/mol	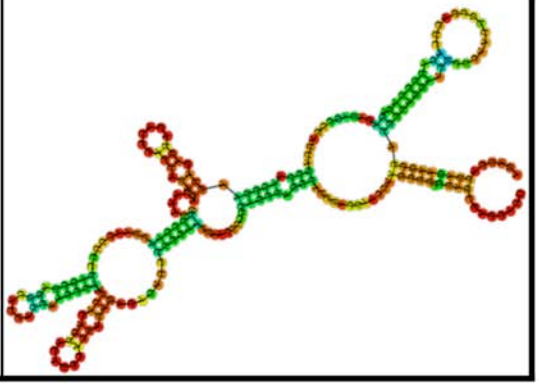

(*Continued*)

VECTOR NAME	SEQUENCE 5' ⇒ 3' (encompassing AUG)	FREE ENERGY	PREDICTED STRUCTURE
**PRL265**	CAGATCACTAGAAGCTATCCTAGGA<br>ACTCTCAATTGCCACTCAGATTCTA<br>GAGACATAGCGCATTAGCCAAGCTA<br>ACTACAGTACATCCACTATAGACAA<br>ATCTTCGAACTAACTTCCTAGCTAG<br>GCATCATCAAGCTAACTACTAACTT<br>CCTAGCTAGGCATCTACTAACTAAG<br>CTATCTCGATCCGTAGATCATACAT<br>AAGCTCTCAGCCGCACGTCTAGCT<br>ACGCATCGGCTAGCTACGCATCAA<br>GCTTCCTAGCCACCATG	-58.30 kcal/mol.	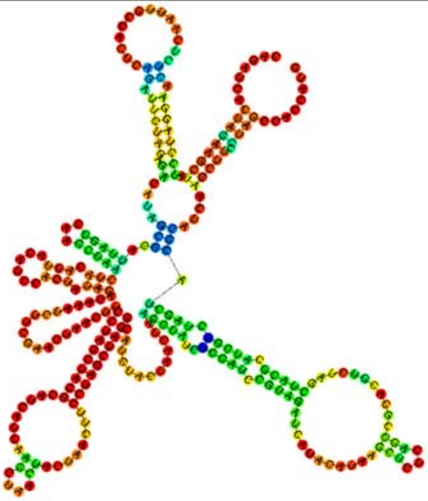
**PRL327**	CAGATCACTAGAAGCTATCCTAGGA<br>ACTCTCAATTGCCACTCAGATTCTA<br>GAGACATAGCGCATTAGCCAAGCTA<br>ACTACAGTACATCCACTATAGACAA<br>ATCTTCGAACTAACTTCCTAGCTAG<br>GCATCATCAAGCTAACTACAGTACA<br>TCCACTATAGACAGATCTTCGAACT<br>AACTTCCTAGCTAGGCATCTACTAA<br>CTAAGCTATCTCGATCCGTAGATCA<br>TACATAAGCTCTCAGCCGCACGTC<br>TAGCTACGCATCGGCTAGCTACGC<br>ATCAGTCCTAGCTGAGCTCGCTAC<br>GTTCACCGCAAAGCTTCCTAGCCA<br>CCATG	-71.95 kcal/mol	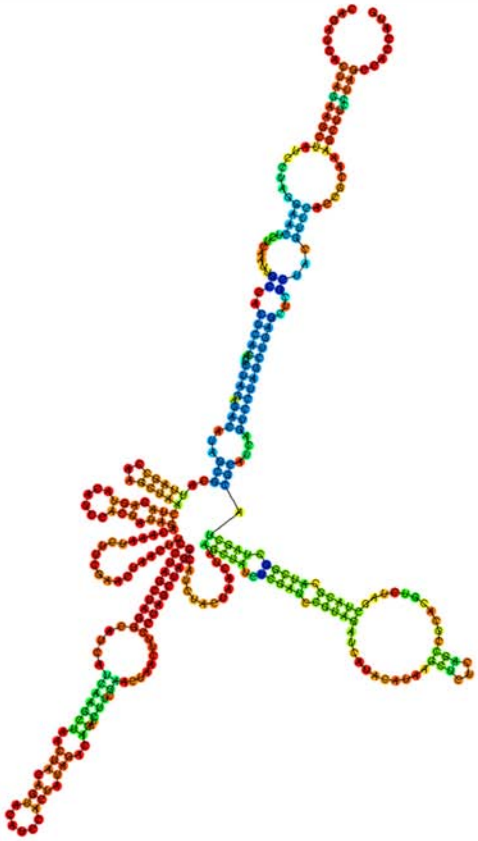

Supplementary Figure S1: (*Continued*) Characteristic of reporter constructs with longer 5'UTRs. For each reporter construct harboring variable 5'UTRs from nucleotides 57 to 327, the vector name (PRL57 to PRL327, in which the number represents the length of the 5'UTR), the sequence of the 5'UTR, the free energy and the predicted structure (RNA fold) are shown. The free energy of the 5'UTR increased from −10.15 kcal/mol for the shorter sequence to −71.95 kcal/mol for the longer one.

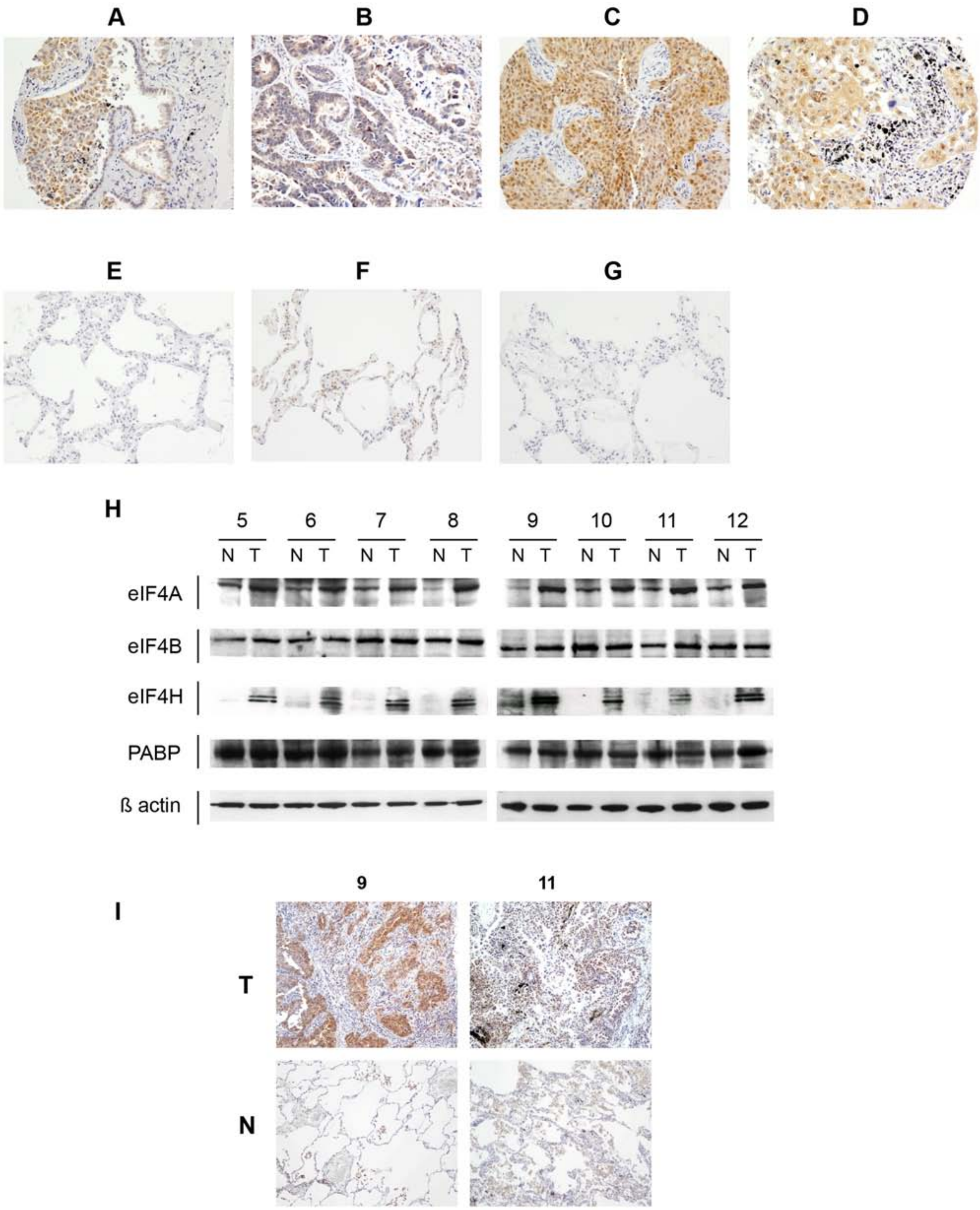

Supplementary Figure S2: eIF4H expression in lung cancer. eIF4H immunostaining results of lung samples obtained from multiple tissue microarray (TMA), corresponding to adenocarcinomas (**A.** and **B.**) epidermoid carcinomas (**C.** and **D.**) or normal lung tissues (**E.** to **G.**)These reveal an intense eIF4H staining in tumoral tissues compared to normal samples. **H.** Western blot analysis of protein lysates prepared from 8 matched samples of lung carcinoma tumors (T) and adjacent non-tumoral tissues (N). Equal amounts of protein from each pair were resolved on 4-20% polyacrylamide gels and immunoblotted with anti-eIF4A, eIF4B, PABP, eIF4H and β-actin (loading control) antibodies. **I.** eIF4H immunostaining of normal (N) and tumoral (T) tissues corresponding to samples 9 and 11 in (H).

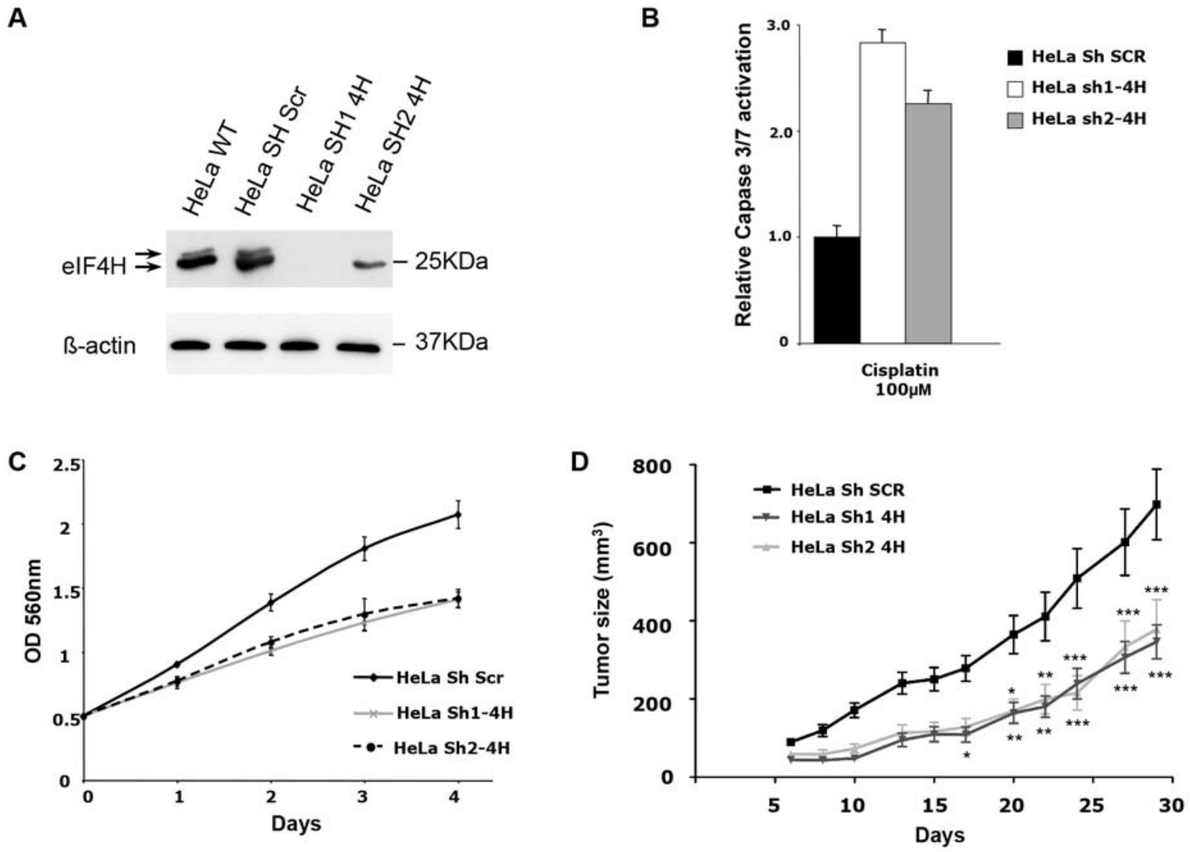

Supplementary Figure S3: *In vitro* and *in vivo* characterization of the effect of eIF4H knockdown in HeLa cells. **A.** Expression analysis of eIF4H and β-actin (loading control) expression in wild type (WT) HeLa cells or in stable clones expressing scrambled shRNA (sh SCR) or eIF4H-targeting shRNA (sh1-4H and sh2-4H). **B.** Caspase 3/7 activity induction after 8 h treatment with cisplatin (100 μM) in HeLa cells expressing eIF4H (sh1 and sh2) or scrambled shRNA. **C.** Cell proliferation of HeLa cells transfected with eIF4H or scrambled shRNA under low serum conditions (0.5%) over 4 days. **D.** Tumor volumes measured at indicated time points after subcutaneous injection of eIF4H-deficient or control HeLa cells into nude mice. Error bars show SEM; $n = 9$.

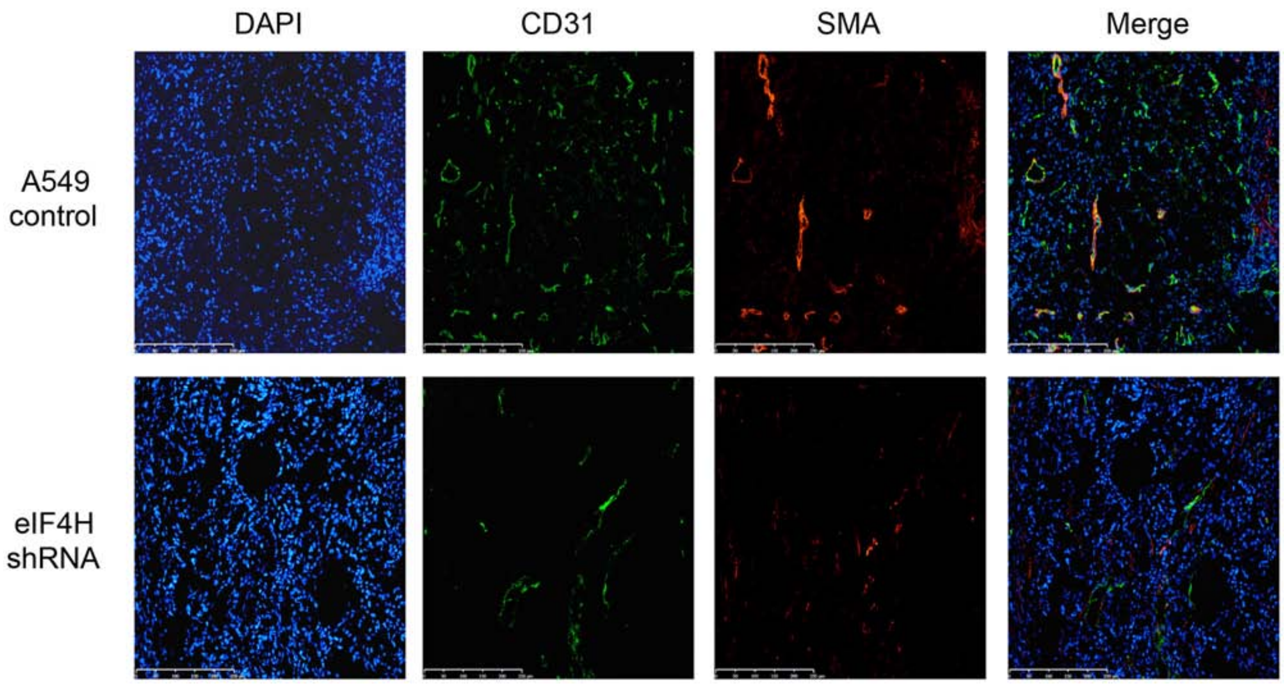

Supplementary Figure S4: eIF4H knockdown inhibits angiogenesis in A549 tumor model. Representative images of control and eIF4H knockdown A549 xenograft stained with anti CD31, a marker to detect endothelial cells (green), anti αSMA a marker for mature pericytes (red), and DAPI (blue). Scale bar = 250 µm. For dual immunofluorescent staining, tumors were incubated with a rat anti-CD31 (1: 50, BD Pharmingen, San Diego, CA, USA) antibody for 2 h followed by incubation with a goat anti-rat Alexa Fluor secondary antibody (1: 250) for 1 h. Then the samples were incubated with a mouse anti-SMa (1:500, Dako Inc) followed by incubation with a goat anti-mouse Alexa Fluor antibody (1: 250) for 1h. Finally, DAPI was added to stain nucleus.

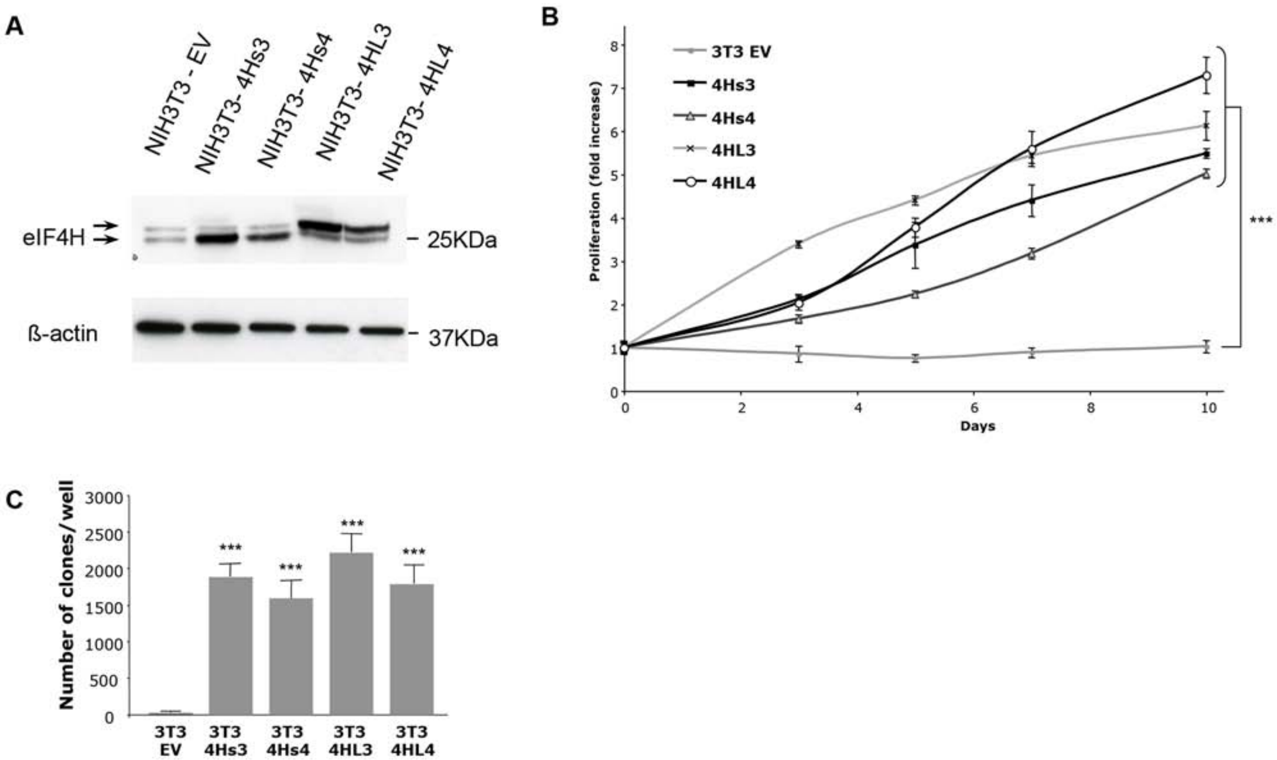

Supplementary Figure S5: Effects of eIF4H overexpression in NIH3T3 cells. A. Expression analysis of two other NIH3T3 stable clones expressing either the eIF4H short isoform (4Hs3 and 4H4) or the long isoform (4HL3 and 4HL4), generated using the "IRES/NEO" gene expression system. The control was provided by NIH3T3 cells stably-transfected with the empty vector (3T3 EV). Loading was normalized to β-actin. **B.** The proliferation of control NIH3T3 cells and stable clones overexpressing the eIF4H isoforms were cultured under low serum conditions (1%) for 10 days. **C.** Colony-formation of eIF4H-transfected NIH3T3 cell lines in soft agar. The mean number of clones in agar was counted after 25 days.

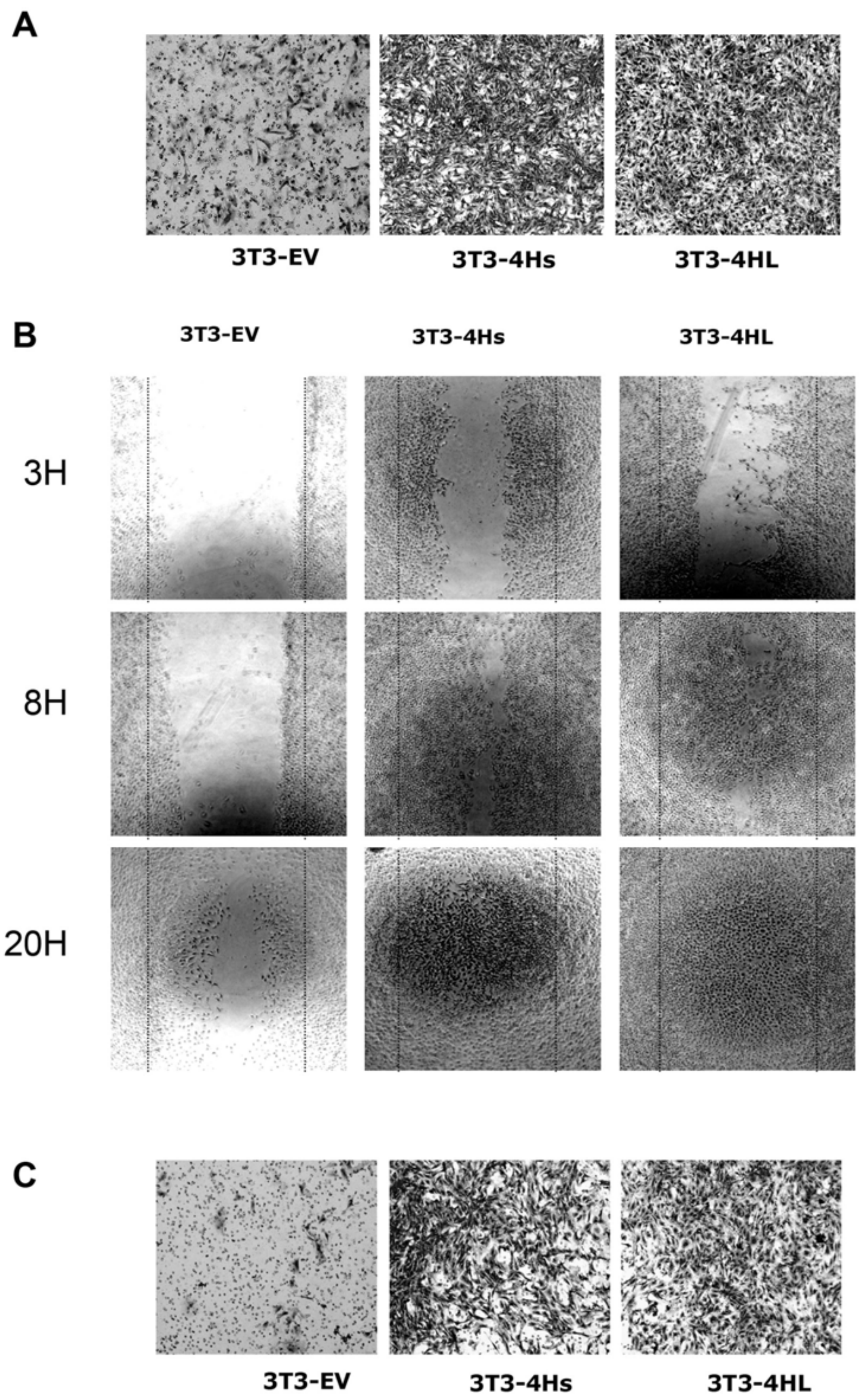

Supplementary Figure S6: Effects of eIF4H overexpression in NIH3T3 cells on cell migration, wound healing and invasion. A. Migratory cells move through the polycarbonate membrane (8µm pore size) toward a chemoattractant (difference in fetal calf serum) underneath the membrane inserts. The migration of clones stably-expressing short (3T3-4Hs) and long (3T3-4HL) eiF4H isoforms were compared with control NIH3T3 cells on the bottom of the polycarbonate membrane. Cells were stained and photographed after 6 hours. **B.** Wound healing is represented by migration in two dimensions. Cells were cultured for 24 hours until a monolayer formed, at which time the inserts were removed to begin the assay. The cells were photographed under a phase-contrast microscope 3 hours, 8 hours and 20 hours after initial wound formation. **C.** The invasive proprieties of clones compared to control NIH3T3 cells were tested through a matrigel membrane. Cells on the bottom of the membrane were stained and photographed at 24 hours.

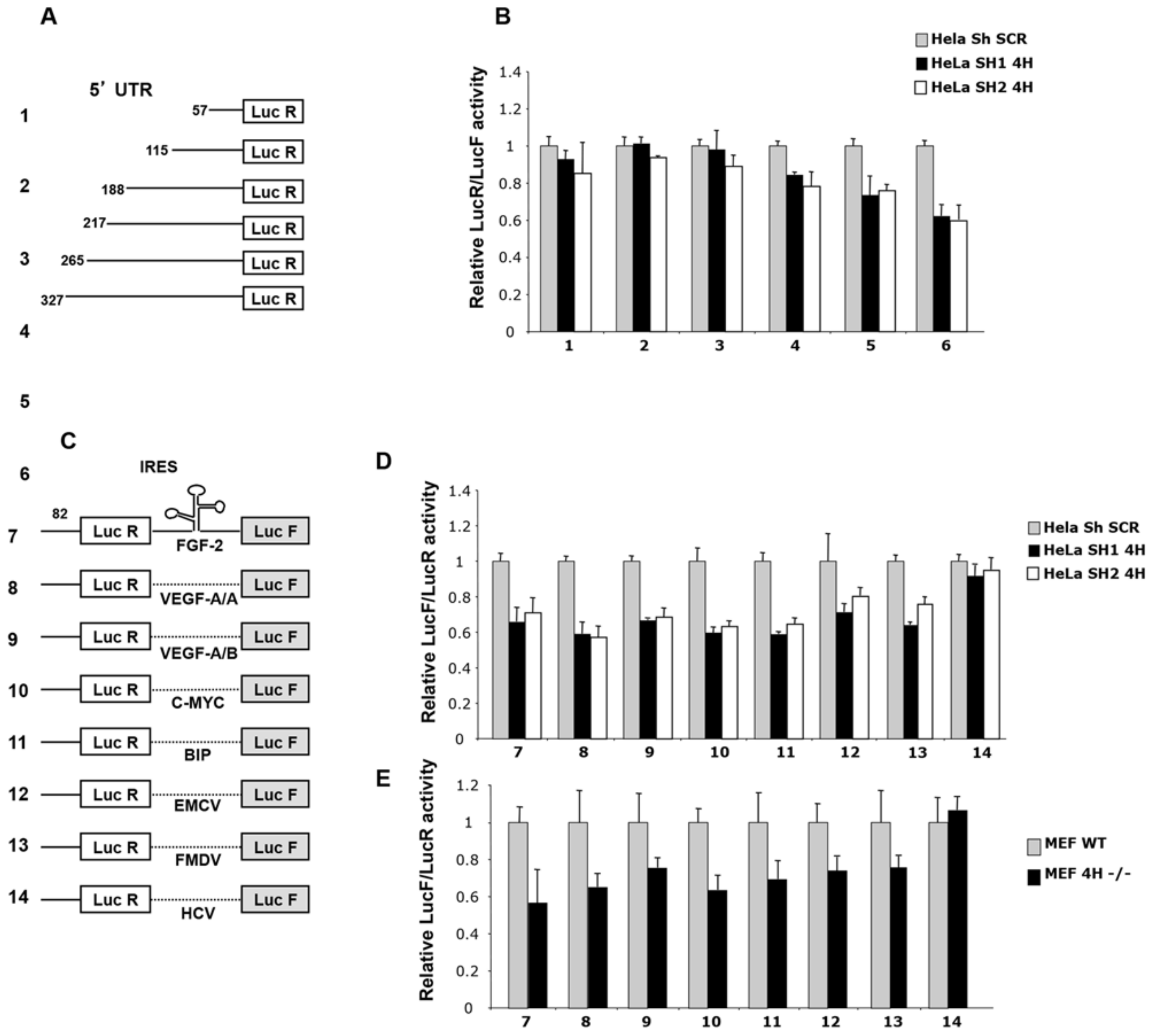

Supplementary Figure S7: Effects of eIF4H depletion on cap- and IRES-dependent translation in HeLa and MEF cells. A. Schematic representation of monocistronic constructs with different 5'UTR lengths. **B.** Ratio of luminescence from the experimental reporter (Renilla) to the control reporter (Firefly; PGL3 from Promega) after co-transfection of HeLa eIF4H knockdown cells (sh1 and sh2), normalized to the HeLa scramble control cells (set as 1). **C.** Schematic representation of bicistronic constructs. IRESs were either viral (EMCV, FMDV, HCV) or cellular (FGF-2, VEGF-A IRESA, VEGF-A IRESB, C-MYC and BIP). **D.** Ratio of luminescence from the IRES-dependent reporter (Firefly) to the cap-dependent reporter (Renilla) in HeLa eIF4H knockdown cells (sh1 and sh2), normalized to the HeLa scramble control cells (set as 1). **E.** Ratio of luminescence from the IRES-dependent reporter (Firefly) to the cap-dependent reporter (Renilla) in MEF eIF4H knockout cells (MEF 4H-/-), normalized to the Wild Type MEF (MEF WT) control cells (set as 1).

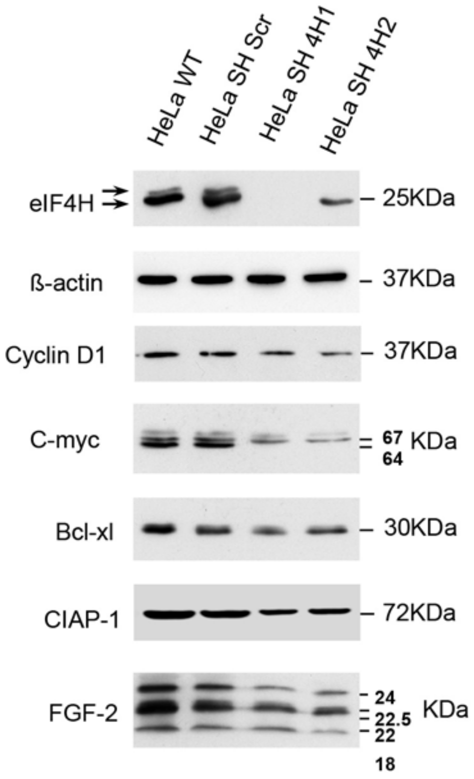

Supplementary Figure S8: Effects of eIF4H depletion in HeLa cells on the regulation of expression of genes involved in proliferation, apoptosis and cellular survival. Western blot analysis of protein lysates prepared from HeLa wild type cells or HeLa cells expressing scrambled (shScr) or eIF4H (sh1-4H, sh2-4H)-targeting shRNAs to compare the expression of proteins encoded by mRNAs harboring complex 5'UTRs. Equal amounts of protein were resolved on 4–20% polyacrylamide gels and immunoblotted with anti-eIF4H, cyclin D1, c-Myc, BCL-xL, CIAP-1, FGF-2 and β-actin (loading control) antibodies.
